# Au nanoparticle-loaded eggshell for electrochemical detection of nitrite

**DOI:** 10.1039/d0ra09892b

**Published:** 2021-01-20

**Authors:** Qi Ding, Liping Cao, Minghuan Liu, Hetong Lin, Da-Peng Yang

**Affiliations:** College of Chemical Engineering and Materials Science, Quanzhou Normal University Quanzhou Fujian 362000 China minghuansdjn@126.com yangdp@qztc.edu.cn; College of Food Science, Fujian Agriculture and Forestry University Fuzhou Fujian 350002 China hetonglin@163.com

## Abstract

Eggshell is an extremely large source of domestic waste and has a huge scientific research potential because of its unique porous hierarchical structure. By converting eggshell waste into valuable functional materials, it can be recycled in many fields. Herein, we envisioned an economical and environmentally friendly conversion method for synthesizing Au nanoparticle loaded eggshell nanocomposites (defined as Au/CaCO_3_ nanocomposites) for the detection of trace amounts of nitrite in oolong tea. Compared with bare electrodes, the prepared Au/CaCO_3_ nanocomposite-based electrodes have obvious electrochemical enhancement behavior. A wide linear response range of 0.01 to 1.00 mM and a relatively low detection limit of 11.55 nM have been obtained in this study. The “turning waste into treasure” transformation strategy not only provides a practical and low-cost method for comprehensive utilization of eggshells as valuable functional materials, but also provides a new approach for sensitive detection of pollutants.

## Abstract 2

The schematic illustrates the preparation process of Au/CaCO_3_ nanocomposites. The bio-derived nanocomposites have successfully been applied for nitrite electrochemical detection.

## Graphical abstract

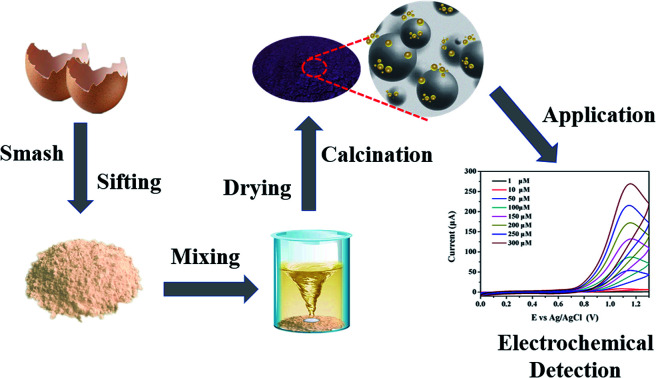

## 1. Introduction

Nitrite is one of the potentially harmful pollutants, and is widely present in the ecological environment, food, industry, and physiological systems. Nitrite is an internationally recognized powerful carcinogen. After people eat foods containing excessive amounts of nitrite, the nitrite can easily combine with secondary and tertiary amines in the food under the acidic conditions of the stomach to transform into carcinogenic *N*-nitramine, which induces cancer.^1–3^ Nitrite is also regarded as the main harmful pollutant in nuclear power plant wastewater, causing serious corrosion behavior. Nitrogen, phosphorus and potassium are vital elements for the growth of vegetation.^4^ Most of the chemical fertilizers used in everyday crops include the above elements. If the amount of nitrogen-containing fertilizer is improperly used, it will degrade and generate a large amount of nitrite to pollute the soil.^4,5^ In addition, nitrite is a widely used preservative because it can effectively inhibit the reproduction of microorganisms.^6–8^ Oolong tea, as a Chinese specialty drink, is well-known worldwide for its unique flavor and rich nutritional value. However, it is susceptible to nitrite contamination during production and processing. In order to protect the health of consumers, it is of great significance to accurately and efficiently detect whether the nitrite ion concentration in tea exceeds the recommended standard. To date, lots of studies have been carried out on the determination of nitrite ions, mainly including spectrophotometry,^9^ gas chromatography,^10^ high performance liquid chromatography,^11^ chemiluminescence,^12^ capillary electrophoresis,^13^ electrochemical methods,^14–18^*etc.* Among them, the electrochemical method has the significant advantages of simple preparation, fast response, low cost, and high sensitivity, attracting widespread attention.^19–23^

As we know, precious metal nanoparticles (Ag, Pd, Pt and Au) have received great attention from many applications for their significant performance. Compared with other precious metal nanoparticles, Au nanoparticles have special advantages because of its high catalysis, excellent optics, and electrical conductivity.^22,24^ Particularly, Au nanoparticles are favored for its outstanding detection performance.^25–29^ However, there are also some limitations in the application of Au nanoparticles. To begin with, small-sized Au nanoparticles are very intolerant to storage due to particle agglomeration.^30^ Besides, Au nanoparticles are expensive and need to be recyclable or easy to recycle without affecting the environment.^31–34^ In order to resolve this problem, it is necessary to build a carrier as a platform to prevent particle aggregation and increase the recovery rate of materials. There are many choices for carriers, such as SiO_2_, graphene, Fe_3_O_4_, as well as CaCO_3_, *etc.* Among them, the naturally-occurring eggshell (CaCO_3_ is the main component) has appealed to many researchers. This is because that eggshell has a unique porous hierarchical structure, which can entrap metal nanoparticles well and effectively prevent particle aggregation,^35,36^ provide abundant 3D active sites, and achieve a high loading rate; secondly, the composition of the eggshell is CaCO_3_ (95%), protein, and a small amount of glycoprotein and proteoglycan.^37–40^ These hydrophilic carboxyl groups and amino groups in proteins can efficiently adsorb Au nanoparticles to form a stable nanocomposite. Most importantly, eggshell ingredients as a waste are very safe and easily available from a wide range of sources in daily life. To fully develop the scientific research value of the eggshell and turn the waste into treasures, in this study, two materials (Eggshell and Au) with excellent properties were combined to construct Au/CaCO_3_ composite nanomaterials and successfully were explored to complete the detection of trace nitrite in oolong tea.

## 2. Experimental

### 2.1 Reagents and instruments

Chloroauric acid (HAuCl_4_, 47.8%) was purchased from Aladdin Chemical Reagent Co., Ltd. (Shanghai, China). Disodium phosphate (Na_2_HPO_4_·2H_2_O, 99.0%) and sodium dihydrogen phosphate (NaH_2_PO_4_·2H_2_O, 99.0%) were purchased from Hengxing Chemical Manufacturing Co., Ltd. (Tianjin, China). Nafion® perfluorinated resin solution (5 wt% in mixture of lower aliphatic alcohols and water) was purchased from Alfa Aesar Chemical Co., Ltd. (Tianjin, China). The eggshells used in this work were obtained from the No. 1 Canteen of Quanzhou Normal University. Oolong tea samples were purchased from Yuting Tea Co., Ltd. (Fujian, China). Graphitized carbon black SPE cartridges were purchased from Anpu Experimental Technology Co., Ltd. (Shanghai, China).

Field emission scanning electron microscope (FE-SEM, Zeiss, Germany). High resolution transmission electron microscope (HR-TEM, Tecnai F30, Netherlands). Selected area electron diffraction (SAED, Tecnai F30, Netherlands). X-ray spectrum detector (EDS, Tecnai F30, Netherlands). Electrochemical workstation (CHI 660E, China). Centrifuge (5810R, Eppendorf, Germany). Ultrasonic cleaning machine (KQ-500DB, Shumei Ultrasonic Instrument Co., Ltd., China). All the water used in the experiment was ultra-pure water (18.2 MΩ cm^−1^), and the glassware involved was washed and dried by ultrasonic cleaning and ultra-pure water.

### 2.2 Pretreatment of eggshell

Wash the fresh eggshell with plenty of water, then submerge it in ultrapure water for 30 min of ultrasonic vibration to remove impurities. Dry the washed eggshells in a constant temperature oven for 5 h to remove moisture, and then put the eggshells into a superfine grinder to pulverize into powder. Finally, the eggshell powder is passed through a 200-mesh sieve to remove large-particle eggshells and obtain relatively fine eggshell powder for subsequent use.

### 2.3 AuNPs modified eggshell powder

Added eggshell powder (0.5 g) to 10 mL of HAuCl_4_ (10 mM). The mixed solution was continuously stirred on a magnetic stirrer for 12 h to fully absorb gold ions by using the porous layered structure of the eggshell. After the completion of the stirring, the suspension was allowed to stand for 1 h to precipitate the eggshell powder, the supernatant was removed with a dropper, and the eggshell fully absorbing gold ions was dried in a constant temperature oven at 60 °C for 1 h. Finally, the sample was placed in a tube furnace in a nitrogen atmosphere and calcined at 500 °C for 2 h at a heating rate of 2 °C min^−1^. Before further analysis, the obtained composite nanomaterials were stored in microcentrifuge tubes at room temperature.

### 2.4 Pretreatment of oolong tea

The tea sample is ground into a powder, and then 1 g of the powder is weighed into a centrifuge tube. Then add 1 mL of nitrite standard solution of different concentrations and 9 mL of acetone solution to each centrifuge tube and shake gently for 10 s. The mixture was then sonicated for 30 min, then the sample was centrifuged for 10 min (1790 × *g*), and then the supernatant was passed through a Carbon-GCB SPE column to remove the pigment. Finally, pour the filtrate into a round-neck flask and use a rotary evaporator to evaporate, and add 1 mL of acetone solution for reconstitution. This solution was added dropwise to 9 mL of phosphate buffer solution for further electrochemical detection. The standard curve of electrochemical detection of nitrite was determined by different concentrations of nitrite solution to calculate the detection limit (LOD) of Au/CaCO_3_.

### 2.5 Data analysis

The detection limit (LOD) of the Au/CaCO_3_ modified electrode is calculated by the following formula:
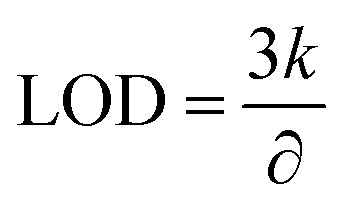


Among them, *k* is the relative standard deviation of the blank, and ∂ is the slope of the calibration curve.

## 3. Results and discussion

### 3.1 Morphology analysis of Au/CaCO_3_ nanocomposite

**Fig. 1:**
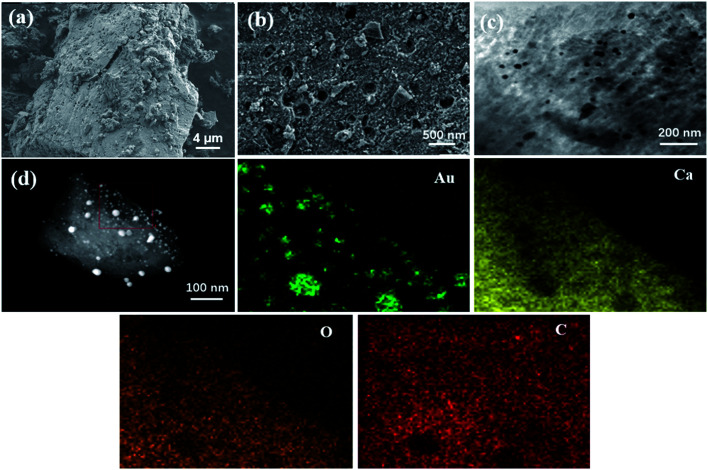
SEM images of (a) the natural eggshell powder; (b) the Au/CaCO_3_ nanocomposites; and (c) HR-TEM images of the Au nanoparticles on eggshell powder, (d) the corresponding EDS elemental mapping images of Au, Ca, O, and C.

The SEM and TEM pictures most intuitively show the surface morphology of Au/CaCO_3_ nanocomposites and natural eggshells. Fig. 1a clearly shows the porous hierarchical structure on the surface of the natural eggshell. The unique porous structure can stably absorb a large amount of Au ions, and the stably adsorbed ions will be reduced to Au nanoparticles *in situ* by high-temperature calcination. Fig. 1b shows the surface morphology of the Au/CaCO_3_ nanocomposite, in which a large number of Au nanoparticles are loaded on the surface of eggshell. Fig. 1c is an HR-TEM image of the nanocomposite material. The particle size and dispersibility of the Au nanoparticles can be further verified, and it is also an important basis for judging whether the loaded Au nanoparticles are agglomerated. In addition, Fig. 1d expresses the elemental composition of the composite nanomaterial. The material is mainly composed of Au, Ca, O and C. The element distribution is shown in the figure. It can be inferred from the mapping image that the Au nanoparticles are uniformly distributed on the surface of CaCO_3_ and are very dense.

### 3.2 Electrochemical performance of Au/CaCO_3_ nanocomposite

**Fig. 2:**
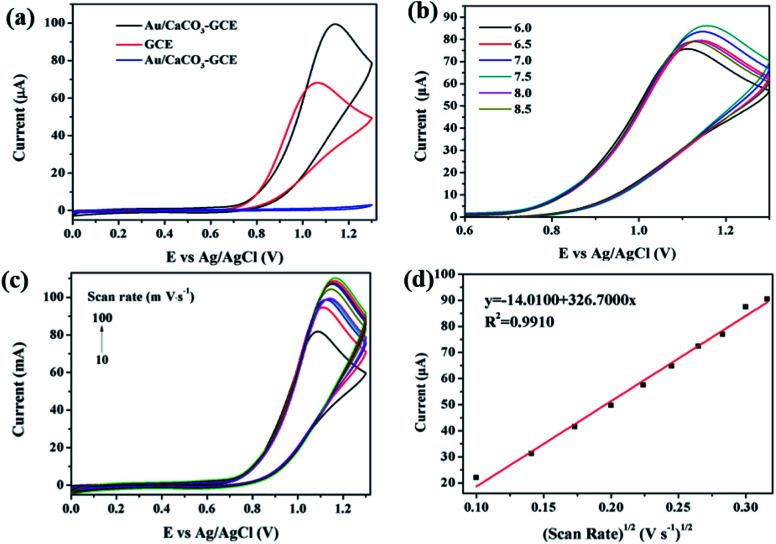
(a) CV curves of bare GCE (red), Au/CaCO_3_-GCE in 100 μM NO_2_^−^ (black) under phosphate buffer (pH = 7.5) and Au/CaCO_3_-GCE in pure buffer (blue) recorded at a scan rate of 50 mV s^−1^; (b) CV responses of Au/CaCO_3_ -GCE in 100 μM NO_2_^−^ at 50 mV s^−1^ under phosphate buffer at varied pH from 6.0–8.5; (c) CV curves of Au/CaCO_3_-GCE in PBS (pH = 7.5) including 100 μM NO_2_^−^ at varied scan rate from 10–100 mV s^−1^; (d) a liner correlation between the peak current and square root of scan rate.

In order to evaluate the electrochemical performance of Au/CaCO_3_ nanocomposites, some data analysis was carried out in this work. Fig. 2a shows the CV curve of the bare GCE of 100 μM nitrite ion, Au/CaCO_3_-GCE and Au/CaCO_3_-GCE recorded at a scan rate of 50 mV s^−1^ in phosphate buffer (pH = 7.5). By comparison, it can be observed that Au/CaCO_3_-GCE can detect obvious characteristic peaks in the phosphate buffer containing nitrite ions, but hardly any peak shape can be captured in the pure buffer. It can be concluded that the characteristic peak detected by CV might correspond to nitrite. Secondly, by comparing the detection of nitrite ions between the bare GCE electrode and Au/CaCO_3_-GCE, it can be observed that the characteristic peak intensity captured by Au/CaCO_3_-GCE is significantly higher than that of the bare GCE electrode. This fully shows that the Au/CaCO_3_ nanocomposite material can significantly enhance the performance of the GCE electrode and effectively capture the signal of trace nitrite ions. The role of Au/CaCO_3_ nanocomposites is mainly reflected in two points: first, Au/CaCO_3_ can increase the surface area of the electrode to promote the adsorption of the analyte. Second, it provides enough Au active sites to accelerate electron transfer, thereby accelerating the overall reaction process.

In order to determine the effect of pH on the electrochemical detection performance of Au/CaCO_3_-GCE, it is necessary to set up a set of control experiments with a variable pH. The contrast CV curve was recorded in 100 μM nitrite ions, and the pH value of the phosphate buffer was fixed at 50 mV s^−1^ in the range of 6.0 to 8.5 during scanning. As shown in Fig. 2b, as the pH increases from 6.0 to 7.5, the corresponding oxidation peak current intensity also increases, and reaches the highest peak at pH 7.5. Subsequently, the pH further increased from 7.5 to 8.5, and the peak oxidation current intensity began to decrease. It can be judged that pH = 7.5 is the best value for detecting nitrite ions.

Then it is necessary to further determine the mechanism of the electrode reaction and explore the influence of the scan rate on the peak current intensity. Fig. 2c shows the CV curve of Au/CaCO_3_-GCE in a fixed pH = 7.5 phosphate buffer. The buffer is added with nitrite ions at a concentration of 100 μM, and the corresponding scan rate interval is set to 10–100 mV s^−1^. The figure intuitively shows that as the scanning rate increases, the intensity of the oxidation peak shows a continuous increase, and the oxidation peak potential gradually shifts. Fig. 2d shows the linear relationship between the peak oxidation current intensity and the square root of the scan rate. They show an obvious positive correlation, the correlation coefficient *R*^2^ = 0.9910, the related linear regression equation can be expressed as:*I*_pa_ (μA) = 326.7100*ν*^1/2^ (mV s^−1^) − 14.0100

The data can powerfully confirm that the Au/CaCO_3_ nanocomposite is a typical diffusion control process for nitrite, and further proves that the nanocomposite is an ideal sensing substance for electrochemical detection of nitrite.

Since the nanocomposite can effectively detect nitrite, it is necessary to analyze the electron rate constant to further quantify its performance. Define the reaction kinetics and electrochemical performance of Au/CaCO_3_-GCE, and calculate the hetero electron rate constant (*K*_s_) of nitrite by the following formula:
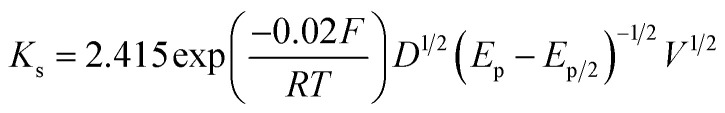
where *E*_p_ is the peak potential, *E*_p/2_ is the potential equal to half of the peak current, *F* = 96 485 is Avogadro's constant (C mol^−1^), *V* is the scanning speed (mV s^−1^), and *D* is the slope (Fig. 2d). By calculation, *K*_s_ = 4.7960 × 10^−2^ cm s^−1^.

### 3.3 The detection limit of Au/CaCO_3_-GCE

**Fig. 3:**
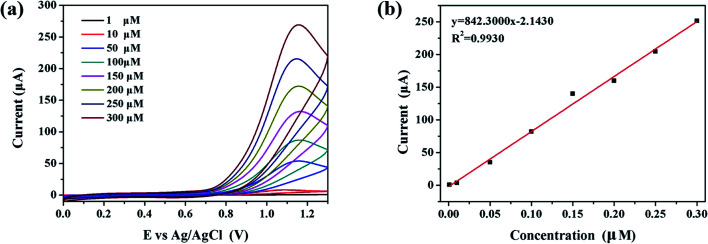
(a) CV response of the Au/CaCO_3_-GCE in pH = 7.5 phosphate buffer at a scan rate of 50 mV s^−1^ under consecutive addition of NO_2_^−^ (concentrations within 1–300 μM), (b) the liner correlations plot of response current *versus* NO_2_^−^ concentrations.

The detection limit is an important indicator to measure the detection performance. In this part of the work, a series of CV detections of nitrite ions were carried out with concentration as the only variable, so as to further the detection limit of Au/CaCO_3_-GCE. Fig. 3a presents the CV curve (1–300 μM) of Au/CaCO_3_-GCE in a phosphate buffer with pH = 7.5 for detecting gradient concentrations of nitrite ions at a scanning speed of 50 mV s^−1^. It can be visually observed in the figure that there is an obvious positive correlation between the concentration of nitrite and the intensity of the characteristic peak. The increase in concentration correspondingly increases the density of nitrite ions per unit volume, and promotes the electrochemical reaction that occurs on the surface of Au/CaCO_3_-GCE, and the resulting potential is thereby increased, resulting in a correspondingly increased CV curve. Fig. 3b shows the linear relationship between response intensity and nitrite concentration, which can be expressed as a linear regression equation through quantitative analysis:*I*_pa_ (μA) = 842.30 μM − 2.1430

The calculated slope is 842.30. Based on the standard formula (LOD = 3*k*/∂), the detection limit can be calculated to be 11.55 nM. In addition, *R*^2^ = 0.9930, indicating that the data has considerable credibility.

### 3.4 Instant current analysis of Au/CaCO_3_-GCE

**Fig. 4:**
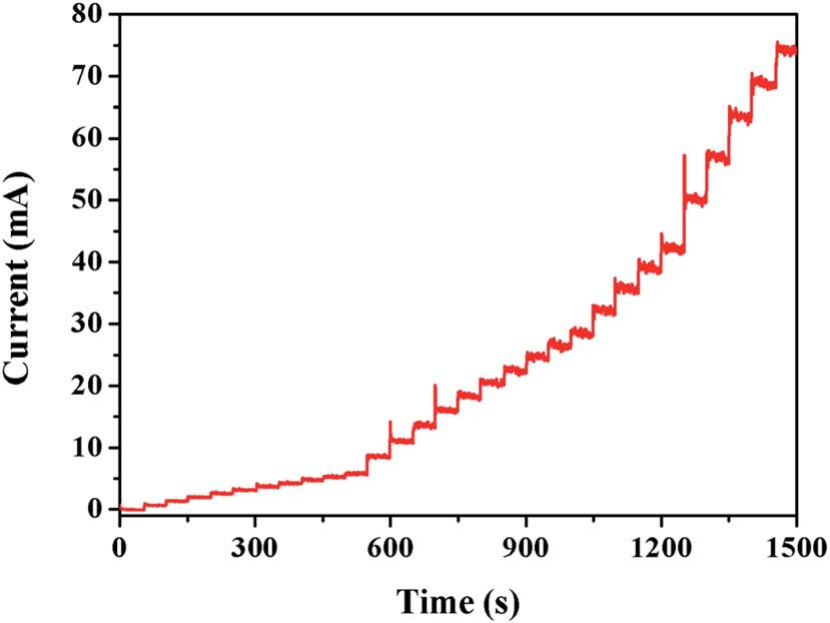
Chronoamperometric graph of Au/CaCO_3_-modified-GCE in 20 mL pH = 7.5 phosphate buffer solution at 1.0 V of the successive additions of NO_2_^−^ at a regular time interval of 50 s.

By analyzing the real-time current graph, it is possible to observe the influence of the current intensity at the moment when the concentration of the analyte changes, thereby reflecting the sensitivity of the Au/CaCO_3_-GCE sensor. When the concentration changes, the current intensity suddenly changes to a steady time is an important indicator of the sensitivity of the detection sensor. Fig. 4 shows the instantaneous ampere intensity change graph of Au/CaCO_3_-GCE at a voltage of 1.0 V when the test substance is continuously added to the supporting electrolyte at an interval of 50 seconds in a 20 mL pH = 7.5 phosphate buffer solution environment. Added 10 mM nitrite to the continuously stirred buffer solution at intervals of 20, 50, and 100 μL showed a typical stepped intensity transition as shown in the figure. The electrode system can respond quickly to each nitrite addition, and the sudden change of current reaches saturation within 5 s and stabilizes quickly, fully demonstrating the excellent sensitivity of the sensor.

### 3.5 The actual samples testing

It has been confirmed that the Au/CaCO_3_-GCE sensor can achieve good detection limits in the standard solution, but whether it can be further promoted still needs to be determined by actual sample detection. In order to evaluate the application of the material in real samples, Au/CaCO_3_-GCE was used to test 4 real samples, including tap water, river water, industrial wastewater, and oolong tea extract. Ensure that the experimental conditions and parameters are the same as above, add 2 μM nitrite ions to each of the four samples, and then use electrochemical methods to detect the recovery rate. Table 1 lists the relevant recovery values of all samples, and the recovery rates of the four samples all present a fairly high level. It is worth mentioning that the detection recovery rate of industrial wastewater exceeds 100%, which shows that the industrial wastewater does contain untreated nitrite, and the rapid detection of nitrite is an urgent task for keeping the safety both environment and human. In summary, Au/CaCO_3_-GCE as a sensor can effectively detect nitrite in oolong tea samples and other actual samples, thereby it might become one of valid tools for food and environmental detection.

**Table 1:** Determination of NO_2_^−^ in different samples at Au/CaCO_3_-GCE by CV^a^

Sample	Added (μM)	Found (μM)	RSD (%)	Recovery (%)
Tap water	2.00	1.89	2.14	94.50
River water	2.00	1.85	1.93	92.50
Industrial waste	2.00	2.25	3.12	112.50
Oolong tea	2.00	1.79	2.47	89.50

a All measurements are an average of *n* = 5.

### 3.6 Performance comparison

Table 2 lists several new electrochemical detection nitrite ion sensors and compares their detection limits. It can be seen intuitively from the table that Au/CaCO_3_-GCE has a lower detection limit than the above-mentioned sensors, which is enough to show that the Au/CaCO_3_-GCE sensor has sufficient application potential. In addition, the sensor is constructed from eggshells, giving it a deeper environmental protection significance.

**Table 2:** An overview on recently reported modified electrodes and their performance compared to the NO_2_^−^ sensors

Electrode	Method	LOD (μM)	Ref.
CuO-GCE	CV	0.400	41
MOF-525	DPV	2.100	42
Co_3_O_4_/RGO-GC	CV	0.140	43
Au–Cu-nanochain/GC	DPV	0.200	44
Cu/MWCNTs/GC	DPV	1.800	45
Au/CaCO_3_-GCE	CV	0.01155	This work

## 4. Conclusions

In this study, the Au/CaCO_3_ nanocomposite was synthesized with eggshell as template by a simple and efficient synthesis method, no toxic reagents and organic solvents were added in the process. The nanocomposite material can be combined with GCE to construct an Au/CaCO_3_-GCE electrochemical sensor for trace detection of nitrite, and the detection limit can reach 11.55 nM. The fabricated Au/CaCO_3_-GCE also shows good performance in the detection of actual samples, and can be applied to the detection of nitrite ions in oolong tea, tap water, industrial wastewater, and river water. The nanocomposite material has the advantages of ultra-small particle size, excellent dispersibility, abundant exposed active sites, convenience, and excellent electronic performance. In addition, the raw material of Au/CaCO_3_-GCE is discarded eggshells. In the current era of advocating green environmental protection, the surplus value of eggshells can be transformed by turning waste into treasure while reducing experimental costs. It is worthy of further development and utilization in near future.

## Conflicts of interest

There are no conflicts to declare.

## Supplementary Material
